# Changes in the Expression Profile of Pyroptosis-Related Genes in Senescent Retinal Pigment Epithelial Cells after Lutein Treatment

**DOI:** 10.3390/cimb45020097

**Published:** 2023-02-09

**Authors:** Barbara Strzalka-Mrozik, Marcel Madej, Natalia Kurowska, Celina Kruszniewska-Rajs, Magdalena Kimsa-Dudek, Jolanta Adamska, Joanna Magdalena Gola

**Affiliations:** 1 Department of Molecular Biology, Faculty of Pharmaceutical Sciences in Sosnowiec, Medical University of Silesia, 40-055 Katowice, Poland; 2 Department of Nutrigenomics and Bromatology, Faculty of Pharmaceutical Sciences in Sosnowiec, Medical University of Silesia, 40-055 Katowice, Poland

**Keywords:** lutein, pyroptosis, oxidative stress, senescent retinal pigment epithelial cells, genes, expression, oligonucleotide microarray, real-time RT-qPCR

## Abstract

Retinal pigment epithelium (RPE) is a specialized structure essential for proper vision, which is constantly exposed to oxidative damage. With aging, this damage accumulates within the RPE cells, causing various diseases, including age-related macular degeneration (AMD). Numerous antioxidant substances are used to prevent this process in humans, including lutein. This study aims to determine the differences in the expression patterns of pyroptosis genes in senescent human retinal pigment epithelial cell line ARPE-19 exposed to lutein. Changes in the expression of pyroptosis-related genes were assessed by oligonucleotide microarrays, and the results were validated by real-time RT-qPCR. The microarray analysis showed seven transcripts were differentially expressed both in the H_2_O_2_-treated cells versus the controls and in the lutein/H_2_O_2_-treated cells compared to the H_2_O_2_-treated cells (FC > 2.0). Depending on the used lutein, H_2_O_2_, or co-treatment of ARPE-19 cells, statistically significant differences in the expression of *TXNIP*, *CXCL8*, *BAX*, and *CASP1* genes were confirmed by the RT-qPCR (*p* < 0.05). A STRING database analysis showed that the proteins encoded by the analyzed genes form a strong interaction network (*p* < 0.001). These data indicate that lutein modulates the expression level of pyroptosis-related genes, which may be useful for the development of new methods preventing pyroptosis pathway activation in the future.

## 1. Introduction

Aging is a natural process that affects all organisms. With age, both physiological and pathological changes are induced in the human body [1]. In the elderly population, age-related macular degeneration (AMD) is the main cause of irreversible blindness and may lead to a reduction in the quality of life [2].

Published data has demonstrated that the retinal pigment epithelium (RPE) plays a key role in maintaining retinal homeostasis and its dysfunction and degeneration is found in many retinal diseases, including AMD [3]. Bruch’s membrane (BrM) thickness and choroid are also integral to the pathogenesis of AMD [4,5]. The most characteristic feature of this pathology is damage to RPE cells due to the formation and deposition of drusen, while its best understood molecular mechanisms are oxidative stress and inflammation [6]. In fact, the retina is exposed to extremely high oxidative stress that increases with age. Large amounts of reactive oxygen species (ROS) are produced in retinal tissue, making it particularly susceptible to oxidative damage [7]. This is because the highest oxygen consumption among human tissues occurs in the retina. Macular and RPE photoreceptors are subjected to constant exposure to light and ultraviolet radiation. There are many photosensitizers in the RPE and photoreceptors. One of them is the easily oxidized polyunsaturated fatty acids (PUFAs), which are present in large amounts in the photoreceptor cell membrane [6,7]. Damage to RPE cells and photoreceptors is particularly associated with exposure to visible light of a short wavelength, as the lens and cornea mainly absorb UV radiation. In addition, a high retinal oxygen partial pressure of 70 mmHg promotes the formation of ROS [6,7]. With age, ROS levels in the aging retina increase, while at the same time, the activity of antioxidant enzymes, such as glutathione, glutathione S-transferases (GSTs), and superoxide dismutase (SOD), leads to oxidative stress, causing damage to the RPE cells and photoreceptors, as well as their programmed death [6,8].

On the one hand, the accumulation of drusen causes inflammation, and inflammatory cells reaching the retina and RPE cells secrete various growth factors, such as vascular endothelial growth factor (VEGF). This contributes to the growth of blood vessels under the RPE layer and choroidal neovascularization (CNV). Ultimately, these pathological processes turn dry AMD into neovascular AMD and lead to central vision loss [8]. Late-stage AMD is characterized by RPE cell death, resulting in photoreceptor dysfunction [9].

It has been suggested that the pathogenesis of AMD involves oxidative and endoplasmic reticulum (ER) stress, impaired autophagy, mitochondrial dysfunction, and inflammation [10]. RPE is the primary pathological site in AMD, but the mechanism of RPE cell death is still unclear.

In addition to the basic process of apoptosis, recent scientific studies have also identified new types of programmed cell death (PCD), which may lead to RPE cell death in AMD. They are pyroptosis, necroptosis, and ferroptosis [8]. The primary step in pyroptosis is the activation of the NLRP3 (NOD-, LRR-, and pyrin domain-containing protein 3) inflammasome, leading to the cleavage of gasdermin D (GSDMD) and the activation of the interleukins IL-1β and IL-18 [11]. Furthermore, inflammasomes play a huge role in the molecular mechanism of pyroptosis [12]. The most widely studied inflammasome is the NLRP3 inflammasome [12]. Its activation is triggered by various cellular and molecular events, such as mitochondrial dysfunction, reactive oxygen species production, ion flow, and lysosome damage [13]. Therefore, the activation of the NLRP3 inflammasome has been detected in various disorders, including macular degeneration.

In 2018, Gao et al. [14] investigated the mechanisms of RPE cell death under prolonged amyloid β-induced inflammasome activity. Strong activation of the inflammasome was evident through increased caspase-1 (CASP1) reactivity and increased levels of IL-1β and IL-18 proteins. In RPE cells, cleavage of caspase-3 (CASP3) and GSDMD, markers of both apoptosis and pyroptosis, was observed. In addition, these cells showed morphological features of pyroptosis, including swelling. This evidence suggests the activation of two death pathways in RPE cells: apoptosis and pyroptosis [14]. In another study from 2020, an in vitro model of AMD involving the induction of retinal pigment epithelium 19 (ARPE-19) damage by amyloid β confirmed the involvement of pyroptosis in RPE cell death [15].

Lutein is one of several compounds from the carotenoid group found in the macula of the retina. It is not produced in the body; therefore, it must be obtained from foods such as broccoli, spinach, kale, peas, lettuce, and egg yolk. Moreover, two other types of carotenoids are also present in the macula, namely zeaxanthin (a stereoisomer of lutein that is also obtained from the diet) and meso-zeaxanthin (a metabolite of lutein that is formed at the macula via metabolic transformation) [16]. These carotenoids exhibit regional dominance in the macula, with lutein present at the highest quantity in the periphery, zeaxanthin in the mid-periphery, and meso-zeaxanthin in the epicenter, respectively [17]. In fact, lutein shows antioxidant activity and functions as a blue light filter, making it one of the greatest allies in the fight against eye diseases. Furthermore, it has anti-inflammatory properties, reduces VEGF expression, and has a limiting effect on the programmed cell death process, which also prevents pathogenesis [17]. The results of several clinical trials suggest an association between the amount of lutein found in food and a reduction in the risk of AMD [17,18]. In recent times, more and more attention has been paid to the analysis of the effect of lutein on the pathogenesis of AMD in experimental studies. Moreover, the effect of lutein on the regulation of pyroptosis signaling pathways is still not fully understood.

Therefore, in this study, we determined the expression profile of pyroptosis-related genes in the human retinal pigment epithelial cell line ARPE-19 exposed to lutein and under conditions of oxidative stress induced by hydrogen peroxide (H_2_O_2_). However, it is worth mentioning that ARPE-19 cells stand as a non-ideal model of primary RPE cells due to some differences between these cells, such as gene expression, phenotype, and possible chromosomal abnormalities, among others [19].

Nevertheless, the ARPE-19 cell line has been used in numerous studies, not least because the ARPE-19 gene expression pattern for apoptosis is quite similar to that of native RPE cells [3,20,21].

## 2. Materials and Methods

### 2.1. Cell Culture Conditions and ARPEs-19 Viability Assessment

Human retinal pigment epithelial cells (ARPE-19, ATCC^®^ CRL-2302; Manassas, VI, USA) were routinely maintained at 37 °C in a 5% CO_2_ incubator (Direct Heat CO_2_; Thermo Scientific, Waltham, MA, USA) with the use of a DMEM-F12 (Dulbecco’s Modified Eagle’s Medium, Lonza, Basel, Switzerland), supplemented with 10% fetal bovine serum (EuroClone). The ARPE-19 cells used in the experiments were from passage 24. Both the cell number and viability were monitored by cell counting in the automated cell counter TC20 (BioRad, Hercules, CA, USA) after staining them with 0.2% trypan blue (Sigma-Aldrich, St. Louis, MO, USA). All the experiments were performed using cells that were approximately 80% confluent.

The MTT (3-[4,5-dimethylthiazol-2-yl]-2,5-diphenyltetrazolium bromide) assay (Sigma-Aldrich, St. Louis, MO, USA) was used to determine the influence of hydrogen peroxide (H_2_O_2,_ Sigma-Aldrich, St. Louis, MO) and lutein (Sigma-Aldrich, St. Louis, MO) on the ARPEs’ viability. The ARPE-19 cells were treated with different H_2_O_2_ concentrations (100; 200; 300; 400 and 500; 1000; 2000 µM) in the medium at 37 °C for 30, 60, and 120 min. For the lutein, the cells were incubated with different concentrations (0.1, 0.5, 1.5, and 10 µM) for 24 h to measure possible cytotoxicity. The controls were left untreated. After respective experimental treatment, cell viability was determined by MTT assay.

MTT (1 mg/ mL) was added to the medium for 3 h (37 °C). After aspirating the MTT, the formazan crystals were dissolved in 100 μL of dimethyl sulfoxide (Sigma-Aldrich Louis, MO, USA). The absorbance was measured at the wavelength of 540 nm with the use of a microplate reader Wallac 1420 VICTOR (Perkin Elmer, Waltham, MA, USA).

### 2.2. ARPE-19 Cells Treatment

The concentrations of H_2_O_2_ and lutein were chosen experimentally and on the basis of available literature data [22]. The ARPE-19 cells were seeded into 6-well culture plates (Thermofisher, Waltham, Ma, USA) at a density of 5 × 10^4^/cm^2^. The next day, the cells were treated with 1.0 µM of lutein for 24 h and 400 µM H_2_O_2_ for 1 h. H_2_O_2_ was added to the cultures 30 min prior to the lutein treatment. In separate cultures, the cells were incubated with lutein or H_2_O_2_ alone at the indicated concentrations and for the indicated time. The untreated cells served as a negative control. Each variant of the experiment was performed in six biological replications.

For the assessment of the mRNA level of the pyroptosis genes, the cells were lysed with the use of TRIzol (Invitrogen Life Technologies, Carlsbad, CA, USA) and then stored at −80 °C for 48 h until further molecular analysis. 

### 2.3. Senescence Assay

The senescence of the ARPE-19 cells was examined using the Senescence Cells Histochemical Staining Kit (Sigma-Aldrich, St. Louis, MO, USA)—senescence-associated β-galactosidase (SA-β-gal), according to the manufacturer’s instructions. SA-β-gal-stained ARPE-19 cells were photographed at 200× magnification. 

The number of SA-β-gal-positive (blue) cells versus the number of total ARPE-19 cells in the ten microscopic fields to calculate the percent of SA-β-gal-positive cells was counted. The values obtained from at least three independent experiments were averaged and the data are presented as the mean ± standard deviation.

### 2.4. Ribonucleic Acid Extraction

The total RNA was extracted using a TRI Reagent (Sigma-Aldrich, St. Louis, MO, USA), according to the manufacturer’s instructions. Purification was performed using the RNeasy Mini Kit (Qiagen Inc., Hilden, Germany, Cat. No. 74106) using columns and DNase I (RNase-Free DNase Set, Qiagen Inc., Hilden, Germany, Cat. No. 79254).

The extracted total RNA was qualitatively and quantitatively evaluated. In the qualitative assessment, the technique of electrophoresis in 1% agarose gel dyed with SimplySafe (EurX, Gdansk, Poland) was used. Quantitative evaluation of the isolated total RNA was performed by spectrophotometric measurement using a MaestroNano MN-913 nano spectrophotometer (MaestroGen Inc., Las Vegas, NV, USA). 

### 2.5. Oligonucleotide Microarray Analysis

Evaluation of the changes in the expression profile of pyroptosis-related genes in the analyzed cells was carried out using the oligonucleotide microarray method. For this purpose, the HG-U133A 2.0 plate set (Affymetrix, Santa Clara, CA, USA) was used in accordance with the manufacturer’s recommendations. 

In order to prepare the matrix for the analysis in the first step, cDNA synthesis was carried out with the SuperScript^®^ Choice System kit (Invitrogen Life Technologies, Waltham, MA, USA). In turn, the BioArray HighYield RNA Transcript Labeling Kit (Enzo Life Sciences, Inc, Farmingdale, NY, USA) was used to obtain biotinylated cRNA. Next, the labeled cRNA were subjected to a fragmentation step using the Sample Cleanup Module kit (Qiagen GmbH, Hilden, Germany). This process was carried out for 35 min at 94 °C. Hybridization of the cRNA to HG-U133A microarrays labeled with a phycoerythrin-streptavidin complex was the final step of the analysis. The scanning was performed using a GeneArray Scanner G2500A (Agilent Technologies, Inc., Santa Clara, CA, USA).

### 2.6. Quantitative Real-Time Polymerase Chain Reaction Assay

The mRNA levels of *TXNIP*, *CXCL8*, *BCL2*, *BAX*, *CASP1*, and *CASP9* genes in the ARPE-19 cells treated with lutein under conditions of H_2_O_2_-induced oxidative stress were assessed using real-time RT-qPCR reactions. The mentioned genes were selected based on a previous microarray analysis and on the available literature data [17,18,19]. In addition, to confirm cellular senescence, the expression of the *p53*, *CDKN1A*, and *CDKN1B* genes were also analyzed. A Sensi-Fast™ reagent kit (Bioline, London, UK) was used for the reactions, as well as pairs of complementary primers (Forward and Reverse), the sequences of which are shown in Table 1. The real-time RT-qPCR reaction was performed using a LightCycler^®^ 480 System apparatus (Roche, Basel, Switzerland) and carried out with the following thermal conditions: reverse transcription 45 °C for 10 min, initial denaturation at 95 °C for 2 min, and then 45 cycles, including: denaturation 95 °C for 5 s, annealing at 60 °C for 10 s, and elongation at 72 °C for 5 s. All the samples were tested in triplicate.

*ACTB* mRNA expression assessment was used as a positive control of amplification. The assessment of the specificity of the reaction was made by analyzing the melting point of the PCR products and electrophoresis in 2% agarose gel.

The mRNA copy number was determined based on a standard curve method described previously by Strzalka-Mrozik et al. [23].

### 2.7. Bioinfromatics Analysis

The data obtained from the oligonucleotide microarrays were analyzed using the PL-Grid Infrastructure (http://www.plgrid.pl/; accessed on 21 September 2022). The GeneSpring 13.0 platform (Agilent Technologies UK Limited, South Queensferry, UK) database was used.

Bioinformatic analysis of the selected pyroptosis-related genes at the protein level was carried out using the STRING online database (https://string-db.org/; accessed on 20 October 2022). An interaction score of >0.4 was selected as the cutoff threshold. The results of protein–protein interactions were presented graphically.

### 2.8. Statistical Analysis

Statistical analysis was performed using STATISTICA 13.3 software (TIBCO Software Inc., Palo Alto, CA, USA). The number of mRNA copies of each gene was recalculated per µg of total RNA. The results of statistical significance were considered those with *p* < 0.05. The qualitative data were presented as mean ± standard deviation (SD) and graphically as a box plot. The W Shapiro–Wilk test was performed to determine the type of distribution. Comparisons of the quantitative variables between the study groups were evaluated using Student’s *t*-test and a one-way ANOVA with a Benjamini–Hochberg correction test. Dunnett’s and Tukey’s post hoc tests were also applied.

## 3. Results

### 3.1. ARPE-19 Viability Assessment

**Figure 1:**
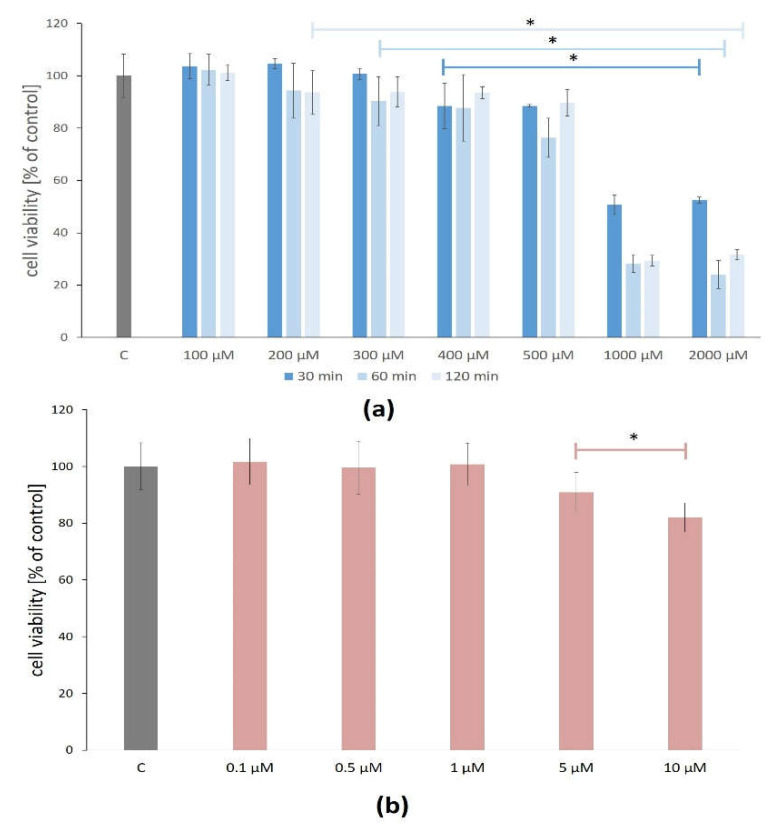
Cell viability of the ARPE-19 cells that were treated with (**a**) H_2_O_2_ for 30, 60, and 120 min., as well as (**b**) treated with lutein for 24 h. Each bar represents the mean ± standard deviation (SD), Dunnett’s test, * *p* < 0.05 versus control (C). Sample size: five wells and three technical replicates for each concetration of H_2_0_2_ and eight wells and three technical replicates for each concetration of lutein.

The H_2_O_2_ concentrations between 100 and 500 μM did not reduce ARPE-19 cell viability below 70% after 30, 60, and 120 min. However, there were statistically significant changes in the ARPE-19 cell viability after exposure to H_2_O_2_ between 400 and 2000 μM after all treatment times (Figure 1a).

The cytotoxicity assay of the lutein effect (in concentrations between 0.1 and 1.0 μM for 24 h) on the viability of the ARPE-19 cells showed no statistically significant differences compared to the control, thus confirming that lutein is not cytotoxic at the tested concentrations for the ARPE-19 cells. The higher studied concentrations (5 and 10 μM) of lutein caused a significant reduction of cell viability on average by 9% and 18%, respectively (Figure 1b).

For further analysis, H_2_O_2_ at the concentration of 400 µM and the treatment time of 60 min and lutein at the concentration of 1.0 µM and the treatment time of 24 h were selected.

### 3.2. Cellular Senescence of ARPE-19 Assessment

**Figure 2:**
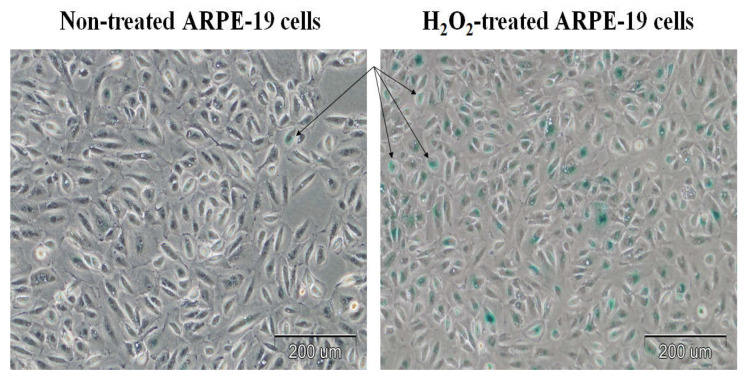
Non-treated and H_2_O_2_-treated ARPE-19 cells stained for senescence-associated β-galactosidase activity. The cells were treated with H_2_O_2_ at a concentration of 400 μM for 60 min. The arrows indicate blue SA-β-gal-positive cells.

**Figure 3:**
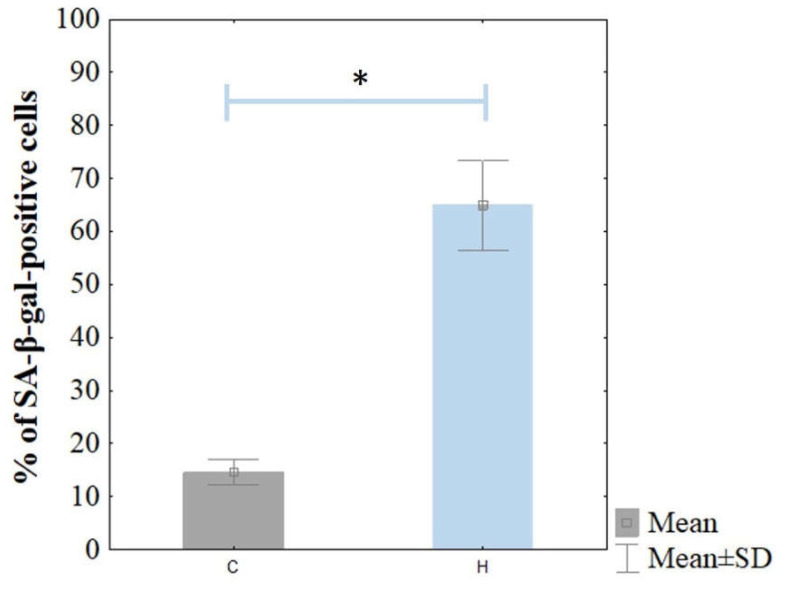
Percentage of SA-β-gal-positive cells in non-treated (C) and H_2_O_2_-treated ARPE-19 cells (H). The bars present mean ± standard deviation (SD); Student’s *t*-test; * *p* < 0.05. The percent of SA-β-gal-positive cells was counted in ten microscopic fields.

Cellular senescence was confirmed based on the significant level of SA-β-gal staining (Figure 2). The ARPE-19 cells were stained for senescence-associated β-galactosidase (SA-β-gal) activity. The H_2_O_2_-treated cells were stained strongly with SA-β-gal compared to the control cells. The percentage of SA-β-gal-positive ARPE-19 cells was 64.9 ± 8.4% in the H_2_O_2_ treated cells, whereas it was 14.6 ± 2.4% in the non-treated ARPE-19 cells (Figure 3).

**Figure 4:**
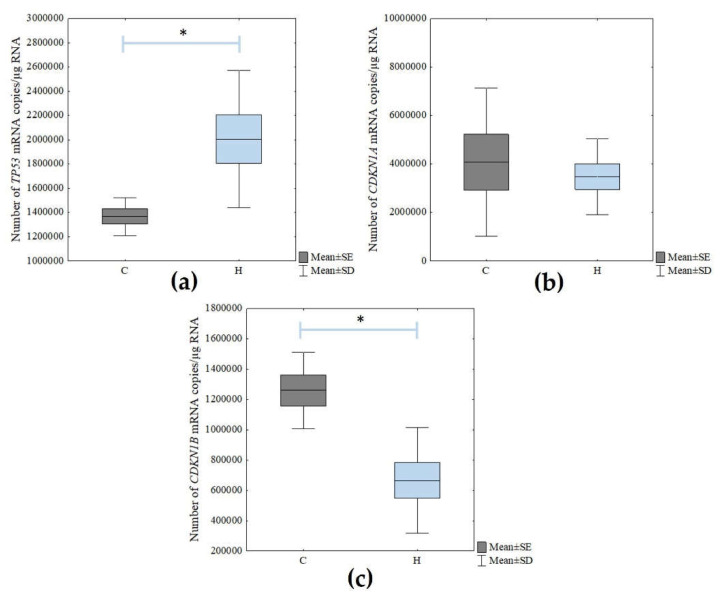
Changes in the mRNA levels of (**a**) TP53, (**b**) CDKN1A and (**c**) CDKN1B genes in non-treated (C) and H_2_O_2_-treated (H) ARPE-19 cells. The box and whisker plots present the mean ± standard error (SE) and standard deviation (SD) of copy numbers per 1 μg of the total RNA; Student’s *t*-test; * *p* < 0.05 versus C. Sample size: three biological and three technical replicates for each test group.

Next, the mRNA levels of the cellular senescence markers (*TP53*, *CDKN1A*, and *CDKN1B*) were determined using the RT-qPCR technique. There was a statistically significant increase in the expression of *TP53* in the H_2_O_2_-treated cells compared to the control cells (Student’s *t*-test; *p* = 0.021). However, there were no statistically significant changes in *CDKN1A* expression. In turn, the level of *CDKN1B* expression decreased statistically significantly due to the action of the hydrogen peroxide (Student’s *t*-test; *p* = 0.003) (Figure 4).

### 3.3. Differential Expression of Pyroptosis-Related Genes Based on Oligonucleotide Microarrays

During the next step of the research, the expression of pyroptosis-related genes was compared using the oligonucleotide microarray technique between the following groups: untreated control ARPE-19 cells (C), lutein-treated ARPE-19 cells (L), H_2_O_2_-treated ARPE-19 cells (H), and lutein- and H_2_O_2_-treated ARPE-19 cells (LH). 

The typing of differentiating genes was performed on a panel of 428 probes for pyroptosis genes obtained from the GeneCards website (https:/www.genecard.org; accessed on 6 October 2022) and from previous studies [24,25]. The selected 239 pyroptosis-related genes are listed in Table S1 in the supplementary materials.

Among them, 128 statistically significant and differentially expressed transcripts were revealed (one-way ANOVA with the Benjamini–Hochberg correction test; *p* < 0.05). 

The nature of the intergroup changes was determined using Tukey’s post hoc multiple comparison test (*p* < 0.05). All the groups were then compared in turn (Table 2).

**Figure 5:**
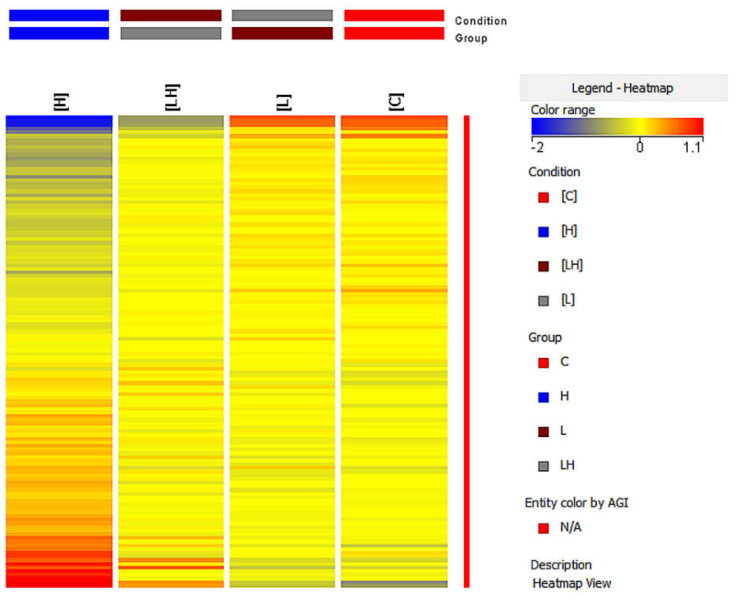
Fluorescence signal intensity maps specific to 128 ID mRNA pyroptosis-related genes specific to the control ARPE--19 cells (C), lutein-treated ARPE-19 cells (L), H_2_O_2-_-treated ARPE-19 cells (H), and lutein-- and H_2_O_2_-treated ARPE-19 cells (LH). Blue—indicates the lowest values and red—indicates the highest values of fluorescence signals.

The differentiation of pyroptosis-related genes between the studied groups was also assessed using the analysis heat map from the GeneSpring XG (Figure 5). Based on the color change of the fluorescence signals, the change in the expression of the studied genes was assessed, where the increase in expression was indicated by a color change toward red and the decrease in expression was indicated by a change toward blue.

A different color pattern was observed in each of the heat maps, thus confirming that the gene expression profile differed in each group.

Subsequently, the genes showing altered expression in the analyzed intergroup were further characterized. The determination of the multiplicity of the gene expression changes was enabled by the fold change parameter (FC), indicating the log2 of the difference in fluorescence signals between the subgroups studied and the direction of the observed change.

Overall, 18 mRNA IDs that had more than a two-fold statistically significant change in expression in at least one pair were determined in this analysis (Table 3).

Of these, seven transcripts were differentially expressed both in the H_2_O_2_-treated ARPE-19 cells versus the controls, and the lutein/H_2_O_2_-treated ARPE-19 cells versus the H_2_O_2_-treated cells (FC > 2.0).

### 3.4. Differential Expression of Pyroptosis-Related Genes Based on Real-Time RT-qPCR

The results obtained from the microarray analysis were also validated using an RT-qPCR reaction. For this purpose, two genes were selected for further analysis: *TXNIP* and *CXCL8,* associated with pyroptosis in ARPE-19 cells, with the highest FC. The *BCL2*, *BAX*, *CASP1,* and *CASP9* genes were also analyzed (selected based on the literature) [26,27,28]. 

**Figure 6:**
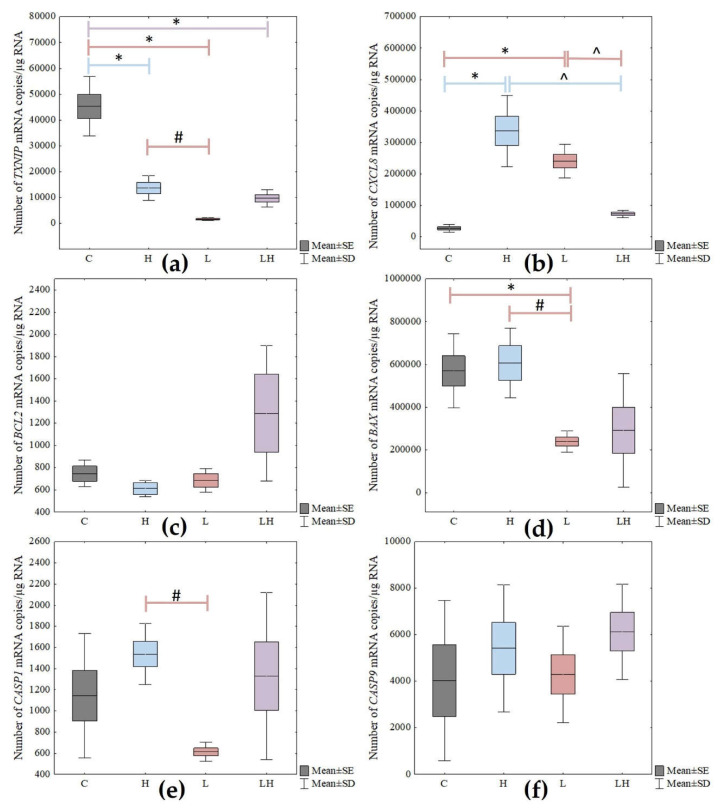
Changes in the mRNA levels of (**a**) *TXNIP*, (**b**) *CXCL8*, (**c**) *BCL2*, (**d**) *BAX*, (**e**) *CASP1*, and (**f**) *CASP9* genes in senescent retinal pigment epithelial cells after treatment with H_2_O_2_ (H), lutein (L), co-treated with lutein and H_2_O_2_ (LH), and non-treated cells (C). The box and whisker plots present the mean ± standard error (SE) and standard deviation (SD) of copy numbers per 1 μg of the total RNA; Tukey’s post hoc test; * *p* < 0.05 versus C; # *p* < 0.05 versus H; ^ *p* < 0.05 versus LH. Sample size—three biological and three technical replicates for each test group.

The level of changes in the expression of pyroptosis-related genes in the ARPE-19 cells exposed to the test compounds, with statistical significance marked, are presented in Figure 6.

Depending on the lutein or H_2_O_2_ used or the co-treatment of cells with both compounds, a statistically significant difference in the genes’ expression of *TXNIP*, *CXCL8*, *BAX,* and *CASP1* (*p* < 0.05) was observed. However, no statistically significant difference was observed for *BCL2* and *CASP9*.

For the ARPE-19 cells treated with lutein (L), a post hoc test showed a statistically significant difference in the *TXNIP* gene expression between the lutein-treated cells versus the control (C) (*p* < 0.001), as well as between the H_2_O_2_ vs. lutein-treated cells (*p* = 0.032).

The analysis of the *CXCL8* gene showed a statistically significant difference between C versus L (*p* < 0.001) and between L versus cells co-treated with H_2_O_2_ and lutein at *p* = 0.001. Additionally, a statistically significant difference was observed in the *BAX* and *CASP1* gene expressions between the L and H_2_O_2_ groups (*p* = 0.027 and *p* = 0.026, respectively). In the case of the *BAX* gene, there was also a change in expression among the control cells and the lutein-treated cells (*p* = 0.025).

In addition, the H_2_O_2_-treated cells showed a difference in the expression of *TXNIP* and *CXCL8* genes at *p* < 0.001 between cells not treated with compounds and those treated with hydrogen peroxide.

Co-treatment with lutein and H_2_O_2_ (LH) of the tested cells resulted in a statistically significant change only in the expression of the *CXCL8* gene as described above, and in the *TXNIP* gene between the C versus LH groups (*p* < 0.001).

### 3.5. Bioinformatic Analysis Using the String Database

**Figure 7:**
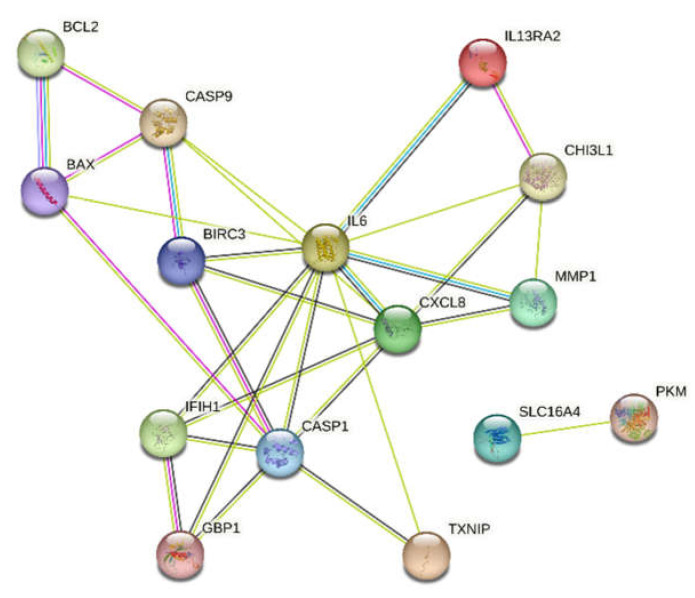
Protein–protein interaction network generated using the STRING database. STRING database—Search Tool for the Retrieval of Interacting Genes/Proteins.

Based on the bioinformatic analysis using the STRING database, the protein–protein interaction results of the selected pyroptosis-related genes were obtained. A network of probable protein–protein interactions consisting of 30 edges and 15 nodes was thus obtained (*p* < 0.001, mean confidence = 0.4). The results of the network of analyzed interactions are presented in Figure 7.

## 4. Discussion

Age-related macular degeneration is a multifactorial disease that occurs in the elderly and its pathogenesis remains largely elusive [1]. It initially affects the RPE, a monolayer of pigmented and polarized central nervous system (CNS) tissue and, over time, leads to a secondary loss of photoreceptor cells [6,7,29].

It should be emphasized that RPE cells play a key role in maintaining retinal homeostasis but are exposed to damaging factors throughout their lives, mainly related to oxidative stress. In addition, structures such as the thickness of Bruch’s membrane or choroid play an important role in AMD pathology [4,5]. Moreover, as a result of aging, more and more oxidative damage occurs in these cells, and consequently, they degenerate [6,7]. Under the influence of adverse factors, these cells can embark on a pathway of programmed death in which various genes and proteins are involved [8,12,30]. In the case of AMD, it has been suggested that it is related to the formation of the NLRP3 inflammasome, which is involved in cleaving gasdermin and setting the cell on the path of pyroptosis [8,12,30]. Many genes are involved in this process, and their proteins have a direct or indirect role in cleaving GSDMD, which is an inducer of pyroptosis [14]. In the course of AMD, inflammation is also observed, mainly due to increased amounts of pro-inflammatory cytokines, chiefly IL-1β and IL-8, which also play a role in leading the cells into the path of pyroptosis [14]. Since oxidative stress greatly affects the level of damage to retinal cells, new methods are needed to prevent the harmful effects of ROS on these cells [6,8]. An example of such a compound, which has natural antioxidant properties, is lutein [31,32]. In addition, it has anti-inflammatory properties and absorbs blue visible light within a 400–500 nm range, which promotes its use in the prevention of AMD [31,32,33]. Moreover, studies suggest that the combination of lutein and zeaxanthin has a positive effect on reducing the incidence of AMD [32,34].

The primary goal of this study was to evaluate changes in the expression of pyroptosis-related genes in senescent ARPE-19 cells after lutein treatment. As the expression pattern of the gene characteristic of apoptosis in ARPE-19 cells is similar to native RPE cells, it was decided that this research model would be used [20,21,22]. The concentration of the compound used did not have a statistically significant cytotoxic effect. Furthermore, cell senescence was induced by culturing the cells in the presence of H_2_O_2_, which was simultaneously assessed by SA-β-gal staining. The percentage of SA-β-gal-positive ARPE-19 cells was shown to be approximately 65%. We considered such a percentage of aged cells sufficient to continue the study. We also evaluated the expression of some genes for cellular senescence—i.e., *p53*, *CDKN1A,* and *CDKN1B* using real-time RT-qPCR [35]. 

Next, we performed a transcriptome analysis using Affymetrix HG-U133A 2.0 oligonucleotide microarrays, which revealed 128 differentiating genes associated with pyroptosis. We narrowed down the obtained genes to FC > 2.0 and obtained seven genes. For further analysis, we selected two of them with the highest FC ratios. In addition, four genes were selected based on the literature data. The selected genes were validated at the mRNA level using a real-time RT-qPCR reaction and in four groups: H_2_O_2_-treated cells, lutein-treated cells, co-treated cells, and non-treated cells, which were the control. In the next step, a bioinformatic analysis was carried out in the STRING database, which revealed the possible protein–protein interactions of the selected genes from oligonucleotide microarrays.

### 4.1. Altered Expression of p53, CDKN1A, and CDKN1B Genes as Additional Confirmation of Cellular Senescence in ARPE-19 Cells

To verify whether the cells actually senesced under the influence of H_2_O_2_, we performed the previously mentioned SA-β-Gal staining assay and evaluated the change in the expression levels of *p53*, *CDKN1A,* and *CDKN1B* genes in non-treated and H_2_O_2_-treated ARPE-19 cells. We observed a statistically significant increase in the expression of the *p53* gene. This gene is mainly involved in cell cycle regulation and is also responsible for the process of apoptosis or cell senescence [35,36]. Zhuge et al. [37], assessing the effect of fullerenol on RPE cells, also used the p53 gene as a marker of senescence. They demonstrated that the compound reduced the expression level of the *p53* gene, which is essential for cell senescence. The mRNA level of the *CDKN1A* (cyclin-dependent kinase inhibitor 1A) gene, also known as *p21^WAF1^*, was not statistically significantly altered under the influence of H_2_O_2_. The role of p21 in senescence was investigated by the team of Dulić et al. [38] using human fibroblasts. The researchers showed that, although this gene is strongly correlated with p53, its levels can vary depending on the number of cell passages. In our study, where the senescent was performed by H_2_O_2_ instead of by passaging, a lack of change in the expression of this gene was found. This may be due to the fact that *p21^WAF1/CIP1^* expression is not maintained in senescent cells and is mainly required for the induction of senescence, as reported by Kumari and Jat [36].

To verify cellular senescence, we also evaluated the *CDKN1B* (cyclin-dependent kinase inhibitor 1B) gene at the mRNA level, which is known as *p27^KIP1^*. It plays a key role in the control of apoptosis, cell cycle, and cell differentiation. The role of p27 in cellular senescence was also investigated by the team of Xu et al. [39] using mesenchymal stromal cells. They demonstrated that the induction of aging by passaging can contribute to a decrease in *p27* levels, which they also linked to the similar expression of β-catenin—a major effector in the Wnt pathway. Also, they observed that with cellular senescent, an increase in the gene expression of *p21* and *p53,* among other genes, occurs while *p27* decreases, which we also demonstrated in our study however, with no change in the *p21* gene expression. Thus, this suggests that the cells were senesced under the influence of H_2_O_2_.

Depending on the compound used, the expression of the genes *TXNIP, CXCL8, BAX*, *BCL2, CASP1*, and *CASP9* in the ARPE-19 cells differed. Liu et al. [40] conducted a similar study in 2017, where, after treating ARPE-19 cells with the same test compounds, they evaluated an increase in the expression of genes by an RT-qPCR reaction, encoding cytokines such as TNF-α, IL-6, and IL-8 associated with inflammatory processes involved in the pathogenesis of AMD. In addition, they showed that hydrogen peroxide and lutein at concentrations >10 µM affect inflammation by upregulating the aforementioned genes [40]. The fact that we carried out the analysis using advanced molecular biology techniques, such as oligonucleotide microarrays, is particularly noteworthy in our work. Due to these techniques, the selected pyroptosis-related genes underwent increased expression after lutein and H_2_O_2_ treatment. In particular, oligonucleotide microarrays and RNA-Seq are the best methods for whole transcriptome gene expression profiling in toxicogenomic studies [41].

### 4.2. Effect of The Tested Compounds on the Expression of TXNIP as a Key Gene Involved in the Process of Pyroptosis

Thioredoxin-interacting protein (TXNIP) plays a key role in the formation of the NLRP3 inflammasome involved in the dysregulation of cell mitochondrial function. It also contributes to the generation of reactive oxygen species [42]. The mechanism of TXNIP is the inhibition of thioredoxin activity, consequently leading to the accumulation of ROS. However, small amounts of thioredoxin are required to refill the total pool of reduced glutathione involved in the antioxidant system [43,44]. In our study, using a real-time RT-qPCR reaction, we found a decrease in the mRNA copy number of the *TXNIP* gene in the ARPE-19 cells after treating them with hydrogen peroxide, lutein, and a co-treatment with both compounds. Therefore, this may suggest that oxidative stress induced by H_2_O_2_ affects the reduction of *TXNIP* gene expression in ARPE-19 cells. In addition, interestingly, lutein, with potential protective effects, caused a similar effect. This is because lutein increases the expression of the *Nrf2* gene, which indirectly contributes to the increase in reduced glutathione by refilling its pool [16]. In addition, the decrease in *TXNIP* gene expression may be related to the lack of appearance of the NLRP3 inflammasome in cells. Cells co-treated with both compounds also led to a decrease in *TXNIP* expression. A similar study was conducted by Ji Cho et al. [44]. They evaluated the effect of oxidative stress on ARPE-19 lines with pLVX-EF1α-IRES-Puro lentiviral vector-induced *TXNIP* gene overexpression. Using an RT-qPCR reaction, they observed that H_2_O_2_ applied as an oxidative stress stimulator reduced the expression level of the *TXNIP* gene, which was also observed in our study. Additionally, it should be noted that until now, the effect of lutein on the *TXNIP* expression on ARPE-19 cells has not been studied. Therefore, our study will introduce new information on the effect of this compound on a key gene, such as *TXNIP*, in the formation of the NLRP3 inflammasome.

### 4.3. Change in CXCL8 Gene Expression as a Key Pro-inflammatory Cytokine Exposed to the Tested Substances

Undoubtedly, one common group of proteins associated with inflammation is cytokines, where interleukins are the largest group [45,46]. Due to their biological functions, they can be either pro-inflammatory or anti-inflammatory. In the case of AMD, a key role is attributed to the C-X-C motif chemokine ligand 8 (CXCL8) gene encoding IL-8, which is classified as a pro-inflammatory cytokine [45,46]. During retinal degeneration, increased amounts of ROS occur, which results in the activation of the immune system associated with inflammation. IL-8, as a common pro-inflammatory cytokine, participates in this process [46]. Our study showed significant changes in the level of mRNA of the *CXCL8* gene in ARPE-19 cells depending on the compound used. 

An increase in the expression of this gene was observed in the H_2_O_2_-treated cells compared to the untreated cells. This suggests, therefore, that in situations of oxidative stress associated with the accumulation of ROS, genes encoding pro-inflammatory cytokines are activated. That fact is supported by a study by Yang et al. [46], which, using molecular biology and western blot techniques, assessed the level of gene expression and cytokine secretion after inducing oxidative stress with H_2_O_2_ and TGF-β2 of ARPE-19 cells. Interestingly, in our study, a similar effect was observed after treating the cells with lutein. This suggests that at this concentration, it may have an effect comparable to hydrogen peroxide. A key observation was noted in the case of ARPE-19 cells treated with H_2_O_2_ to induce oxidative stress and simultaneous treatment with lutein at a concentration of 1.0 µM. In this case, there was a significant decrease in the mRNA copy number of the *CXCL8* gene. This suggests that lutein abolishes the effect of H_2_O_2_, thus inhibiting the formation of ROS. To date, the effect of lutein on the *CXCL8* level in ARPE-19 cells has not been studied, but Kim et al. [47] conducted a similar study. In a study on human gastric epithelial cell lines treated with H_2_O_2_ conducted earlier in the experiment, they examined the simultaneous effects of β-carotene and lutein on IL-8 expression and secretion [47]. The molecular analysis using real-time RT-qPCR and enzyme-linked immunosorbent assay (ELISA) proved that the combination of the two compounds has an inhibitory effect on H_2_O_2_, affecting the reduction of IL-8 secretion by impacting the suppression of the transcription factor NF-κB [47]. A decrease in *CXCL8* expression under the influence of lutein was also observed in our study on ARPE-19 cells.

### 4.4. Effect of the Studied Compounds on APRE-19 Cells in Reference to the Genes Involved in the Process of Apoptosis

Some of the important genes involved in the process of cell death by apoptosis are the *BAX* (BCL2-associated X protein) and *BCL2* (B-cell lymphoma) genes [48]. BAX is a pro-apoptotic protein that can induce either apoptosis or pyroptosis during stress factors, such as chemotherapy. The activation of the BAK/BAX pathway causes activation of the caspase cascade due to the occurrence of the mitochondrial outer membrane permeabilization (MOMP) effect [49]. The pathway to which the cell will be activated subsequently depends on BAX-activated caspases, which will turn the cell on the pyroptosis pathway when gasdermin E (GSDME) is cleaved [49]. In our own studies conducted on ARPE-19 cell lines, the level of *BAX* gene expression changes depending on the compound used, and was investigated using oligonucleotide microarrays, as well as real-time RT-qPCR reaction. It was observed that the mRNA copy number of this gene did not change statistically significantly when H_2_O_2_, a strong stimulator of oxidative stress, was applied, as well as when the combination of lutein and H_2_O_2_ was used. Interestingly, a statistically significant decrease in the amount of the *BAX* gene was noted in the lutein-treated ARPE-19 cells, which may suggest that this compound indirectly affects the process of apoptosis or pyroptosis in cells, but this requires further study. Hu et al. [49] carried out a similar study, however, on cells of different neoplastic lines. Using flow cytometry and western blot analysis, they demonstrated that the application of chemotherapy induces the occurrence of pyroptosis in cells through the BAK/BAX-caspase 3-GSDME pathway. Interestingly, they also concluded that single proteins of BAK and BAX are able to independently contribute to the development of pyroptosis. In our own study, using molecular biology techniques, we found that the level of *BAX* gene expression in ARPE-19 cells does not change statistically significantly.

BCL2, on the other hand, belongs to a group of anti-apoptotic proteins, whose function is to bind to the pro-apoptotic proteins BAX and BAK, limiting their oligomerization and contributing to the inhibition of apoptosis [50]. In a study conducted by Shi et al. [50] on cells of the neoplastic lines HT-29 and THP-1 using immunoblotting and immunoprecipitation, the researchers found that the BCL2 protein negatively affected the NLRP3 inflammasome. In addition, they showed that the protein inhibits the cleavage of GSDMD by caspases, resulting in a reduction in the entry of cells into the pyroptosis pathway [48]. In our own study on ARPE-19 cells treated with H_2_O_2_ and lutein, no changes in *BCL2* gene expression were observed. This suggests, therefore, that these compounds do not significantly affect the mRNA level of this gene, and thus, probably do not affect the development of pyroptosis in cells associated with the *BCL2* gene.

### 4.5. Expression of Caspase-1 and Caspase-9 mRNA in ARPE-19 Cells Treated with Test Compounds

Caspases, whose main function is to cleave GSDMD, certainly play an important role in the development of pyroptosis. This leads to the onset of pyroptosis, which is characterized by the occurrence of inflammation preceded by the activation of interleukins, mainly IL-1β and IL-18 [51,52,53]. A key function in this footnote is played by caspase-1 (CASP1), which is activated by inflammasomes [51]. In a study conducted by Tseng et al. [54] on eye tissue sections taken from patients with geographic atrophy or neovascular AMD, as well as the ARPE-19 cell line, they investigated the effect of lysosomal destabilization on NLRP3 inflammasome activation. Through the immunohistochemical technique and an RT-qPCR reaction, they proved that there is an increase in the NLRP3 inflammasome in AMD. In addition, lysosomal destabilization caused the activation of caspase-1, which induced a pyroptosis effect on the cells. In our own study on ARPE-19 cells, an increase in *CASP1* mRNA was observed in cells treated with H_2_O_2_ compared to the controls, but the increase was not statistically significant. Interestingly, a decrease in *CASP1* expression was observed in cells treated with lutein, which may indicate that this compound has a protective effect on the cells, as it decreases the expression of caspase-1, which is key to inducing the pyroptosis effect.

Caspase-9 (CASP9) also plays an important role in the process of programmed cell death [52]. It is a cysteine-aspartate protease whose role is to initiate intrinsic apoptosis. In addition, changes in *CASP9* expression levels are observed in many diseases including retina-related eye diseases, and caspase-9 is also indirectly associated with CASP3/GSDME [52,55,56]. In a study conducted by Lu et al. [57] on lung cancer samples from patients, as well as cell lines and an animal model, they evaluated the effects of selected antitumor drugs on various forms of cell death. They used immunohistochemical methods, electron microscopy, and analysis at the protein level using western blot techniques. In their experiment, they concluded that caspase-9 participates in the stimulation of apoptosis in cells, which in turn activates the caspase-3/GSDME pathway and leads cells into the pyroptosis pathway [57]. Our studies on ARPE-19 cells treated with hydrogen peroxide and lutein did not observe significant statistical changes in the level of *CASP9* expression. This suggests, therefore, that the tested compounds probably do not affect the entry of cells into the apoptosis or pyroptosis pathway mediated by caspase-9. However, this requires further research.

It should be noted that although ARPE-19 cells are similar to normal RPE cells in terms of morphology and the expression of some genes, they have limitations [57,58]. In a study by Ablonczy et al. [59], the researchers showed that ARPE-19 cells have reduced expression of some key markers that characterize RPE cells in vivo. APRE-19 cells have lower expression of the RPE65 marker and cellular retinaldehyde-binding protein (CRALBP), components that are essential for the normal pattern cycle in RPE cells. In addition, there are different strengths of cellular connections compared to normal cells in vivo [59]. However, the researchers suggest that the cells of the ARPE-19 line have a morphology more similar to aging RPE or pathological cells. To elaborate, they could be used in studies of the effects of exogenous substances to reverse pathologic changes. Therefore, these differences should be taken into account when planning an experiment, and the appropriate model for the study should be chosen [58,59].

In summary, through the techniques of molecular biology, we demonstrated the effect of the studied compounds on the expression levels of pyroptosis-related genes (Table S2 in the supplementary materials). 

Knowledge of the effect of lutein on the expression of genes in ARPE-19 cells may be useful for the development of new methods to prevent the activation of the pathway of programmed death called pyroptosis, which may result in a reduction in the number of events associated with AMD in the future. It should be noted that our results were obtained using in vitro experiments, which cannot be directly compared with in vivo studies.

## 5. Conclusions

Our results suggest that exposure to lutein of senescent retinal pigment epithelial cell line ARPE-19 changes in the level of transcriptional activity of pyroptosis genes. Investigating the influence of lutein on the transcriptional activity of these genes may provide additional information about the molecular mechanisms of their action. Moreover, lutein may have an effect on the processes activated by pyroptosis-related genes in different ways depending on the presence or absence of H_2_O_2_ induced oxidative stress.

It should be noted, however, that the effect observed for the cell line when tested in vitro may not be the same for an organ response in vivo. Moreover, our results should also be confirmed at the protein level. Because the number of studies on pyroptosis-related genes in AMD is limited, more research is needed.

## Figures and Tables

**Table 1:** Primer sequences for amplification of *TXNIP*, *CXCL8*, *BCL2*, *BAX, CASP1, CASP9, TP53, CDKN1A*, and *CDKN1B* mRNAs.

Gene	Oligonucleotide Sequence	Amplimer Length (bp)	Tm (°C)
*TXNIP*	Forward: 5′CAGCCAACAGGTGAGAATGA3′ Reverse: 5′TTGAAGGATGTTCCCAGAGG3′	104	83.1
*CXCL8*	Forward: 5′CTCTAACTCTTTATATAGGAATT3′ Reverse: 5′GATTGATTTTATCAACAGGCA3′	203	81.2
*BCL2*	Forward: 5′GATTGTGGCCTTCTTTGAG3′ Reverse: 5′GTTCCACAAAGGCATCC3′	164	85.8
*BAX*	Forward: 5′CCTGTGCACCAAGGTGCCGGAACT3′ Reverse: 5′CCACCCTGGTCTTGGATCCAGCCC3′	99	84.4
*CASP1*	Forward: 5′CAACTACAGAAGAGTTTGAGG3′ Reverse: 5′AACATTATCTGGTGTGGAAG3′	92	82.0
*CASP9*	Forward: 5′CTCTACTTTCCCAGGTTTTG3′ Reverse: 5′TTTCACCGAAACAGCATTAG3′	148	84.4
*TP53*	Forward: 5′ACCTATGGAAACTACTTCCTG3′ Reverse: 5′ACCATTGTTCAATATCGTCC3′	99	83.7
*CDKN1A*	Forward: 5′ CAGCATGACAGATTTCTACC3′ Reverse: 5′CAGGGTATGTACATGAGGAG3′	200	88.4
*CDKN1B*	Forward: 5′ AACCGACGATTCTTCTACTC3′ Reverse: 5′ TGTTTACGTTTGACGTCTTC3′	133	84.7
*ACTB*	Forward: 5′TCACCCACACTGTGCCCATCTACGA3′ Reverse: 5′CAGCGGAACCGCTCATTGCCAATGG3′	295	88.4

bp—base pair; Tm—melting temperature.

**Table 2:** The number of probe set identifiers (IDs) differentiating the examined transcriptome groups from each other.

Tukey’s Post-Hoc Multiple Comparison Test				
Transcriptome Group	IDs [L]	IDs [H]	IDs [LH]	IDs [C]
IDs [L]	–	112	28	3
IDs [H]	16	–	98	119
IDs [LH]	100	30	–	28
IDs [C]	125	9	100	–

[L]—lutein-treated ARPE-19 cells; [H]—H_2_O_2_-treated ARPE-19 cells; [LH]—lutein and H_2_O_2_-treated ARPE-19 cells; [C]—control; white—the number of mRNA IDs that differentiated the transcriptome groups; dark grey—the number of mRNA IDs in the comparative differentiation that is unrepresentative; light grey—the number of mRNA IDs that were representative of the differentiation of the transcriptome groups being tested; sample size: three biological replicates for each test group.

**Table 3:** Characteristics of pyroptosis-related genes, which had more than a two-fold statistically significant change in their expression in at least one pair.

Probe ID	Gene Symbol	*p* Value	FC			
			[H] vs. [C]	[H] vs. [LH]	[L] vs. [C]	[LH] vs. [C]
**201008_s_at**	***TXNIP***	*p* < 0.001	**↓** **6.90**	**↓** **2.35**	↓1.02	↓2.94
**201009_s_at**	***TXNIP***	*p* < 0.001	**↓** **5.56**	**↓** **2.12**	↓1.03	↓2.62
**201010_s_at**	***TXNIP***	*p* < 0.001	**↓** **5.73**	**↓** **2.07**	↓1.02	↓2.77
202269_x_at	*GBP1*	*p* < 0.001	↑2.06	↑1.19	↑1.03	↑1.73
202270_at	*GBP1*	*p* < 0.01	↑2.11	↑1.06	↓1.11	↑1.99
**202859_x_at**	***CXCL8***	*p* < 0.001	**↑** **8.57**	**↑** **3.42**	↑1.39	↑2.51
**203685_at**	***BCL2***	*p* < 0.001	**↑** **2.30**	**↑** **2.40**	↓1.11	↓1.04
**204475_at**	***MMP1***	*p* < 0.001	**↑** **3.50**	**↑** **3.57**	↓1.05	↓1.02
205207_at	*IL6*	*p* < 0.001	↑2.00	↑1.30	↑1.17	↑1.49
205234_at	*SLC16A4*	*p* < 0.01	↓2.05	↓1.83	↓1.17	↓1.12
206172_at	*IL13RA2*	*p* < 0.001	↑2.11	↑1.96	↓1.11	↑1.08
209395_at	*CHI3L1*	*p* < 0.01	↑1.71	↑2.17	↓1.09	↓1.27
209396_s_at	*CHI3L1*	*p* < 0.01	↑1.65	↑2.05	↓1.12	↓1.25
210538_s_at	*BIRC3*	*p* < 0.01	↑2.15	↑1.95	↑1.03	↑1.10
**211506_s_at**	***CXCL8***	*p* < 0.001	**↑** **9.28**	**↑** **4.12**	↑1.26	↑2.25
213700_s_at	*PKM*	*p* < 0.001	↓2.02	↓1.11	↓1.15	↓1.81
214657_s_at	*NEAT1*	*p* < 0.01	↓3.58	↓1.76	↓1.36	↓2.04
219209_at	*IFIH1*	*p* < 0.001	↓2.15	↓1.81	↑1.00	↓1.19

[C]—control; [L]—lutein-treated ARPE-19 cells; [H]—H_2_O_2_-treated ARPE-19 cells; [LH]—lutein- and H_2_O_2_-treated ARPE-19 cells; FC—fold change (FC > 2.0); ↑, ↓—higher and lower gene expression; ID—the probe set identifier; bold—genes that exhibit differential expression in the H_2_O_2_-treated ARPE-19 cells versus controls, and lutein-/ H_2_O_2_-treated ARPE-19 cells versus the H_2_O_2_-treated cells. Sample size: three biological replicates for each test group.

## Data Availability

The datasets used and/or analyzed during the current study are available from the corresponding author upon reasonable request.

## Supplementary Materials

The following supporting information can be downloaded at: https://www.mdpi.com/article/10.3390/cimb45020097/s1. Table S1: Pyroptosis-related genes selected for oligonucleotide microarrays analysis; Table S2: The classification of selected pyroptosis-related genes with a description of their function based on the STRING database.
