# Supplementary material for: Changes in the Expression Profile of Pyroptosis-Related Genes in Senescent Retinal Pigment Epithelial Cells after Lutein Treatment
Source: Curr Issues Mol Biol. 2023 Feb 9;45(2):1500–18. doi: 10.3390/cimb45020097 (PMC9955508; doi:10.3390/cimb45020097)
Supplement: Supplementary file 1 [file cimb-45-00097-s001.zip › cimb-2157598-supplementary.pdf]

Supplementary materials

Table S1. Pyroptosis-related genes selected for oligonucleotide microarrays analysis.

Gene symbol					
ABL1	*CASP6*	*EZH2*	*IRF2*	*PANX1*	*SUZ12*
ACE2	*CASP8*	*FADD*	*IRF3*	*PARP1*	*TFAM*
ADORA1	*CASP9*	*FGF21*	*JUN*	*PDCD6IP*	*TFAP2A*
ADORA2A	*CD14*	*FMR1*	*KCNQ1OT1*	*PECAM1*	*TLR2*
ADORA2B	*CDC37*	*FNDC4*	*KIF23*	*PGF*	*TLR3*
ADORA3	*CDK9*	*FOXO3*	*KLF3-AS1*	*PKM*	*TLR8*
AGER	*CEBPB*	*FOXP3*	*LRPPRC*	*PKN2*	*TNF*
AIM2	*CEP55*	*FPR2*	*LY96*	*PLCG1*	*TNFRSF13B*
ALK	*CHI3L1*	*GBP1*	*LYST*	*POLA1*	*TP53*
ANXA1	*CHMP1A*	*GJA1*	*MALT1*	*POLA2*	*TP63*
ANXA2	*CHMP2A*	*GLMN*	*MAP3K7*	*POP1*	*TRAF6*
APAF1	*CHMP2B*	*GPER1*	*MAPK14*	*PPARG*	*TREM1*
APIP	*CHMP3*	*GPX4*	*MDM2*	*PRDM1*	*TREM2*
APOE	*CHMP4A*	*GSDMB*	*MEFV*	*PRF1*	*TRIM21*
APOL1	*CHMP6*	*GSDMD*	*MEG3*	*PRIM1*	*TRIM24*
ASIC1	*CHMP7*	*GSK3B*	*MELK*	*PRIM2*	*TRIM31*
ATF6	*CLEC5A*	*GZMA*	*METTL3*	*PRKACA*	*TRPM2*
ATG3	*CPTP*	*GZMB*	*MKI67*	*PRMT5*	*TUBB6*
ATG7	*CRTAC1*	*HDAC6*	*MMP1*	*PRTN3*	*TXNIP*
BAK1	*CSNK1A1*	*HMGB1*	*MST1*	*PTEN*	*UBE2D2*
BAX	*CTSG*	*HSP90AA1*	*MYD88*	*PTGS2*	*UBE2D3*
BCL2	*CTSV*	*HSP90AB1*	*NAIP*	*PTPN11*	*UBR2*
BECN1	*CXCL8*	*HTRA1*	*NEAT1*	*PYCARD*	*UCP1*
BHLHE40	*CYCS*	*HUWE1*	*NEDD4*	*RAB5A*	*USF2*
BHLHE41	*DDX3X*	*ICAM1*	*NFE2L2*	*RBBP4*	*USP24*
BIRC2	*DHX9*	*IFI16*	*NFKB1*	*RBBP7*	*USP47*
BIRC3	*DNMT1*	*IFIH1*	*NFS1*	*RIPK1*	*USP8*
BNIP3	*DNMT3A*	*IKZF1*	*NINJ1*	*SCAF11*	*UTS2*
BRCC3	*DNMT3B*	*IL13*	*NLRP1*	*SDHB*	*VCAM1*
BRD4	*DPEP1*	*IL13RA2*	*NLRP2*	*SEC22B*	*VDR*
BSG	*DPP8*	*IL18*	*NLRP3*	*SERPINB1*	*VIM*
BST2	*DRD2*	*IL18BP*	*NLRX1*	*SEZ6L2*	*VPS28*
BTK	*DUOX1*	*IL1A*	*NOD1*	*SIRT1*	*VPS4B*
CAMP	*E2F4*	*IL1B*	*NOD2*	*SLC16A4*	*VTN*
CAPN1	*EED*	*IL1RN*	*NOS1*	*SNIP1*	*XIST*
CARD8	*EGFR*	*IL32*	*NOS2*	*SQSTM1*	*YWHAE*
CASP1	*ELANE*	*IL36G*	*NR1H2*	*STAT3*	*YWHAZ*
CASP3	*ELAVL1*	*IL6*	*OSM*	*STK4*	*ZBP1*
CASP4	*EPHA2*	*IRAK3*	*P2RX7*	*STXBP2*	*ZNF532*
CASP5	*ERP44*	*IRF1*	*PAK2*	*STXBP3*	

Table S2. The classification of selected pyroptosis-related genes with a description of their function based on the STRING database.

Gene	Function	RT-qPCR results		
		[H] *vs.* [C]	[L] *vs.* [C]	[LH] *vs.* [C]
TXNIP	Role in the formation of the NLRP3 inflammasome; may act as an oxidative stress mediator by inhibiting thioredoxin activity or by limiting its bioavailability	↓*	↓*	↓*
CXCL8	It is released from several cell types in response to an inflammatory stimulus; involved in neutrophil activation	↑*	↑*	↑
BCL2	Regulates cell death by controlling the mitochondrial membrane permeability; may attenuate inflammation by impairing NLRP1-inflammasome activation, hence CASP1 activation and IL1B release	Ns	Ns	Ns
BAX	Can induce either apoptosis or pyroptosis during stress factors and depending on caspases; Can induce pyroptosis via BAK/BAX-caspase 3-GSDME pathway	Ns	↓*	Ns
CASP1	Main function is to cleave GSDMD; activated by inflammasome	↑	Ns	Ns
CASP9	Cysteine-aspartate protease involved in apoptosis; indirectly associated with CASP3/GSDME pathway which can result in pyroptosis in cells	Ns	Ns	Ns

*[C]—control; [L]—lutein-treated ARPE-19 cells; [H]—H2O2-treated ARPE-19 cells; [LH]—lutein- and H2O2-treated ARPE-19 cells; ↑, ↓—higher and lower gene expression; *—statistical significance (p<0.05), Tukey's post hoc test; Ns—not statistically significant; RT-qPCR—reverse transcription quantitative real-time polymerase chain reaction; TXNIP—thioredoxin-interacting protein; CXCL8—C-X-C motif chemokine ligand 8; BCL2—B-cell lymphoma; BAX—BCL2-associated X protein; CASP1—caspase-1; CASP9—caspase-9; NLRP3—NOD-, LRR- and pyrin domain-containing protein 3; NLRP1—NOD-, LRR- and pyrin domain-containing protein 1; IL1B—interleukin 1 beta; BAK—BCL2 homologous antagonist killer; GSDME—gasdermin E; CASP3—caspase 3; GSDMD—gasdermin D; STRING database—Search Tool for the Retrieval of Interacting Genes / Proteins.*
